# Association of Decreased Serum BDNF With Restless Legs Syndrome in Parkinson's Disease Patients

**DOI:** 10.3389/fneur.2021.734570

**Published:** 2021-10-26

**Authors:** Yi-xian Huang, Qi-lin Zhang, Cai-li Huang, Wen-qi Wu, Jia-wei Sun

**Affiliations:** ^1^Department of Neurology, The Second Affiliated Hospital of Soochow University, Suzhou, China; ^2^Department of Psychiatry, Johns Hopkins University School of Medicine, Baltimore, MD, United States

**Keywords:** Parkinson's disease, brain-derived neurotrophic factor, restless legs syndrome, serum, biomarker

## Abstract

**Objective:** To objective of the study was to investigate whether serum brain-derived neurotrophic factor (BDNF) levels are associated with the severity of restless legs syndrome (RLS) in Parkinson's disease (PD).

**Methods:** A total of 249 PD patients with (*n* = 53) and without RLS (*n* = 196) and 326 age-matched controls were included in this study. All the serum BDNF levels of the participants were measured. The International Restless Legs Syndrome Study Group Rating Scale (IRLSSG-RS) was administered for the severity of RLS. The severity of PD patients were assessed by the Unified PD Rating Scale (UPDRS) and the Hoehn and Yahr (H-Y) stage.

**Results:** The prevalence of RLS was significantly higher in PD patients (21.3%) than in the controls group (7.4%) (*p* < 0.05). The IRLSSG-RS score in PD patients with RLS (16.25 ± 5.24) was significantly increased than in controls with RLS (12.08 ± 3.99) (*p* < 0.01). The serum BDNF levels were significantly decreased in PD patients with RLS than in PD patients without RLS, controls without RLS, and controls with RLS (*p* < 0.001). BDNF levels were negatively associated with IRLSSG-RS in both PD patients with RLS and controls with RLS group (both *p* < 0.01). Multiple regression analysis confirmed that in either PD with RLS or controls with RLS group, BDNF was an independent contributor to IRLSSG-RS (both *p* < 0.01).

**Conclusions:** Decreased serum BDNF levels may be involved in the pathophysiology of RLS in PD, suggesting that it may serve as a potential blood biomarker of diagnostic value for RLS in PD.

## Introduction

Restless legs syndrome (RLS) is a sensorimotor disorder characterized by extreme discomfort due to irresistible urges to move the legs (1). The symptoms worsen during rest, mainly at night, and the symptoms can be at least partially and temporarily relieved by physical activity (2, 3). Parkinson's disease (PD) is one of the most common neurodegenerative diseases with motor symptoms and non-motor abnormalities (4–6). Various previous studies have found that the prevalence rate of RLS was higher in PD patients than in the general population, affecting 10–50% of parkinsonian patients (7–11). Several studies have also suggested that RLS is a possible preclinical marker of PD (12). Indeed, most genetics and pathology studies point to a strong association between RLS and PD (13).

Although the etiology of PD patients with RLS is still poorly understood, pathophysiological and pharmacotherapeutical studies suggest that dopamine (DA) dysfunction plays an important role in both PD and RLS pathophysiology (14, 15). Brain-derived neurotrophic factor (BDNF) is a critical member of the neurotrophin family, which is involved in the mediation of the neuronal survival and development (16). It is a protein mostly expressed in the central nervous system, especially in the striatum, substantia nigra, and ventral tegmental area, which contains a major portion of the dopaminergic (DAergic) cell groups of the ventral midbrain (17–19). Several lines of evidence reveal that the pleiotropic activities of BDNF play a vital role in the survival and maintenance of DAergic neurons, and lower BDNF expression in substantia nigra is associated with dopamine neuronal loss in PD patients compared with that in controls (20, 21). Consistently, a lot of improving treatments of PD are accompanied by BDNF enhancement (22, 23). These studies suggest that the reduced BDNF levels may be associated with pathological alterations of the DAergic neurons in the substantia nigra. In view of the reduced DAergic neurons playing an important role in the pathogenesis of RLS, future studies should explore the relationship between BDNF and RLS in PD patients.

Therefore, the goal of this study was to explore the association between BDNF and RLS in PD patients. To achieve this goal, we recruited a comparatively large sample of PD patients (*n* = 249) and matched controls (*n* = 326), and tried to establish correlations between serum BDNF levels and RLS assessed by the International Restless Legs Syndrome Study Group Rating Scale (IRLSSG-RS) (24). We hypothesized that serum BDNF levels would be decreased in PD patients with RLS, and reduced BDNF levels might be associated with the severity of RLS in PD.

## Materials and Methods

### Participants

Baseline characteristic and clinical data were enrolled from patients with idiopatic PD (125 men and 124 women) at the Department of Neurology of the Second Affiliated Hospital of Soochow University. All patients with PD met the following inclusion criteria: (1) age 40–80 years old, Han Chinese; (2) all patients were diagnosed independently by two neurologists, according to the United Kingdom PD Society Brain Bank Criteria (25). Patients with atypical parkinsonism such as the multiple system atrophy, progressive supranuclear palsy, and vascular or secondary parkinsonism were excluded. (3) Patients >2 years diagnosed with PD; (4) receiving stable doses of L-Dopa/carbidopa administered at least three times per day for at least 4 weeks before the initial screening visit; (5) patients treated with dopamine agonists, monoamine oxidase-B inhibitors and other antiparkinsonian drugs, antidepressant, anxiolytics, and antiepileptic drugs (gabapentin, pregabalin, etc.) were excluded; (6) patients with secondary restless leg syndrome (pregnancy, iron deficiency anemia, kidney disease, diabetes, peripheral neuropathy, etc.) were excluded; (7) at least 8 years of education; (8) without severe cognition impairment; Mini-Mental State Examination (MMSE) score below 24 out of 30 points were excluded; (9) all patients provided written informed consent and can be tested for IRLSSG-RS.

Additionally, 326 age-matched control subjects (age, 61.61 ± 9.67 years) with no history of parkinsonism were recruited from the local community at the same period and matched for age, sex, and education. All subjects were Chinese Han population. Subjects with secondary restless leg syndrome (pregnancy, iron deficiency anemia, kidney disease, diabetes, peripheral neuropathy, etc.) were excluded. No subjects were administered with dopamine agonists and other antiparkinsonian drugs, antidepressant, anxiolytics, and antiepileptic drugs (gabapentin, pregabalin, etc.).

After making the diagnosis of RLS, PD patients were divided into two groups: those PD with RLS and those PD without RLS. In the same way, the controls were divided into two groups: those controls with RLS and those controls without RLS.

### Clinical Symptom Assessment

Demographic information including age, sex, educational, marriage, alcohol, and smoking behavior as well as PD clinical characteristics were collected by a member of the research staff. Additional information was collected from available medical records. We assessed severity of the PD patients using the Hoehn and Yahr (H-Y) stage (26) and the Unified Parkinson's Disease Rating Scale (UPDRS), as previously described (27), which were evaluated only during “on” condition. For each treated patient, the levodopa equivalent dose (LED) was calculated as previously reported (28).

According to the criteria of Jankovic et al. (29), based on the UPDRS, we subdivided PD patients into the tremor-dominant (TD) and postural instability/gait disorder (PIGD) subtypes.

### Restless Legs Syndrome Diagnosis and Assessment

According to the four essential criteria described by the International Restless Legs Syndrome Study Group (IRLSSG) criteria (30, 31), all subjects were diagnosed with definite RLS. The severity of RLS was measured by the IRLSSG rating scale (IRLSSG-RS) as described previously (24), consisting of 10 items, graded into five severity categories from 0 to 4 (total score range 0–40). Generally, RLS was divided in groups with mild (0–10 points), moderate (11–20 points), severe (21–30 points), or very severe (31–40 points) (24). IRLSSG-RS was completed in “on” states.

### Blood Sampling and Serum Brain-Derived Neurotrophic Factor Examination

We collected peripheral blood from each subject between 8:00 and 9:00 a.m. prior to clinical assessment and following an overnight fast. Blood samples were transferred into anticoagulant-free tubes and then set aside for clot formation at room temperature for 1 h. The serum was obtained by centrifugation at 1,620 relative centrifugal force for 15 min and then kept frozen at −80°C until assay. The BDNF assay was performed using a solid-phase, sandwich enzyme-linked immunosorbent assay (ELISA) kit (DuoSet; R&D Systems, Minneapolis, MN, USA), according to the instructions of the manufacturer. This assay was fully described in our previous report (32). All measurements were assayed in duplicate, and the mean was calculated. All blood samples from patients and controls were simultaneously analyzed by a research assistant blind to the clinical situation in the same ELISA templates. The variability among measures was monitored by utilizing four samples with a known concentration of BDNF. The mean inter- and intra-assay variation coefficients were ~8%.

### Statistical Analysis

The sociodemographic and clinical characteristics between groups were compared using analysis of variance (ANOVA) for continuous variables, and chi-square for categorical variables. We tested the homogeneity of variances by using the Kolmogorov–Smirnov one-sample test. For variables (serum BDNF level, H-Y, UPDRS score, LED, and IRLSSG-RS) that did not display a normal distribution, the groups were compared with non-parametric Mann–Whitney *U*-test (for two groups) or Kruskal–Wallis test (for >2 groups). Where there was a significance in ANOVA, the effect of sex, age, education, BMI, alcohol, smoking status, and clinical variables was tested by adding these variables to the analysis model as covariates. To determine the diagnostic accuracy, a receiver operating characteristic (ROC) curve was constructed to determine the area under the curve (AUC) and the values of sensitivity and specificity with 95% confidence interval (CI), and Youden index was determined (sensitivity + specificity − 1.0) to find the optimal cutoff value. Correlation among variables was assessed by the Pearson's correlation coefficient and Spearman's rank correlation coefficient. Bonferroni corrections were applied to each test to adjust for multiple testing. A multivariate regression analyses was used to assess the association of IRLSSG-RS with BDNF while adjusting for potentially confounding demographic and clinical variables, including sex, age, education, marriage, BMI (body mass index), alcohol, smoking status, age of onset, duration of disease, H-Y stage, and UPDRS score. Effect sizes (0.2 = small effect, 0.5 = medium effect, 0.8 = large difference effect) were calculated using the Cohen's d method for the two-way comparisons and represented the mean difference, in standard deviation units, between the groups of interest. The SPSS statistics (version 19.0, IBM Corp., Armonk, NY, USA) was utilized for all statistical analyses. All data are expressed as mean and standard deviation (mean ± SD). All *p*-values were two-tailed with a significance level set at 0.05.

## Results

### Demographic and Clinical Characteristics of Participants

The demographic characteristics of PD patients along with controls are shown in Table 1. The patients had a mean age of 62.0 ± 9.3 years and a mean duration of disease of 4.3 ± 2.4 years. The prevalence rate of RLS was 21.3% in PD patients, compared with only 7.4% in the matched controls group, representing a highly significant difference (*p* < 0.05; Table 1). The IRLSSG-RS score in PD patients with RLS was significantly higher than that in controls with the RLS group (PD with RLS, 16.25 ± 5.24; controls with RLS, 12.08 ± 3.99; t = −3.457, *p* = 0.001; Table 1). There was no significant difference in sex, age, education, alcohol, smoke, BMI, age of onset, disease duration, LED, H-Y, and UPDRS score among controls without the RLS group, controls with the RLS group, and PD patients with and without the RLS groups, respectively (all *p* > 0.05). There was no significant association between BDNF levels and sex, age, education, alcohol, smoke, BMI, age of onset, disease duration, LED, H-Y, and UPDRS score among controls without the RLS group, controls with the RLS group, and PD patients with and without the RLS groups, respectively (all *p* > 0.05). Based on the UPDRS score, which was 37–42, and the mean H-Y stage, which was 1.5–1.8, a mild to moderate degree of PD in this study is suggested.

**Table 1:** Clinical characteristics and demographic details of Parkinson's disease patients with RLS and without RLS and controls.

**Variables**	**Control without RLS**	**Control with RLS**	**PD without RLS**	**PD with RLS**	***P*** **-value^a^**
	**(***n*** = 302)**	**(***n*** = 24, 7.4%)**	**(***n*** = 196)**	**(***n*** = 53, 21.3%)**	
Sex (M/F)	168/134	11/13	102/94	23/30	0.339
Age (years)	61.61 ± 9.31	61.58 ± 13.69	61.72 ± 9.17	62.81 ± 9.94	0.867
Education (years)	9.10 ± 1.94	9.17 ± 1.86	9.16 ± 1.92	9.32 ± 2.06	0.886
Marriage					
Married	283/302	21/24	183/196	46/53	0.099
Single	0/302	1/24	1/196	1/53	
Divorced	10/302	1/24	6/196	3/53	
Widowed	9/302	0/24	6/196	4/53	
BMI (Kg/m^2^)	23.91 ± 2.12	23.63 ± 2.18	23.77 ± 1.71	23.46 ± 2.08	0.441
Smoker/non-smoker	45/257	3/21	23/173	5/48	0.616
Alcohol/non-alcohol	39/263	4/20	21/175	6/47	0.793
Age of onset (years)	NA	NA	57.47 ± 8.25	58.09 ± 8.67	0.632^b^
Disease duration (years)	NA	NA	4.25 ± 2.33	4.72 ± 2.74	0.214^b^
H-Y	NA	NA	1.56 ± 0.78	1.75 ± 0.94	0.126^b^
UPDRS score	NA	NA	37.47 ± 14.05	41.34 ± 15.53	0.083^b^
LED (mg/d)	NA	NA	410.15 ± 181.09	448.11 ± 190.03	0.182^b^
IRLSSG-RS	NA	12.08 ± 3.99	NA	16.25 ± 5.24	0.001^c^
BDNF (pg/ml)	4779.18 ± 771.59	3502.63 ± 904.08	3814.57 ± 835.38	2604.40 ± 1011.22	0.000^b^,^c^

*Data are presented as the mean ± standard deviation (mean ± SD)*.

*BDNF, brain-derived neurotrophic factor; BMI, body mass index; H-Y, the Hoehn and Yahr staging scale; IRLSSG, International Restless Legs Syndrome Study Group; LED, levodopa equivalent dose; PD, Parkinson's disease; RLS, restless legs syndrome; UPDRS, Unified Parkinson's Disease Rating Scale*.

a *Parameters were analyzed with analysis of variance using Bonferroni as post-hoc test in the case of normal distribution of data. For variables that did not display a normal distribution, data were compared with the Kruskal–Wallis test for comparison of multiple groups, or Mann–Whitney U-test for comparison of two groups. Sex was analyzed using χ^2^-test*.

b *Differences were found between PD with RLS vs. PD without RLS*.

c *Differences were found between PD with RLS vs. control with RLS*.

### Serum Brain-Derived Neurotrophic Factor Levels in Parkinson's Disease Patients With and Without Restless Legs Syndrome

**Figure 1:**
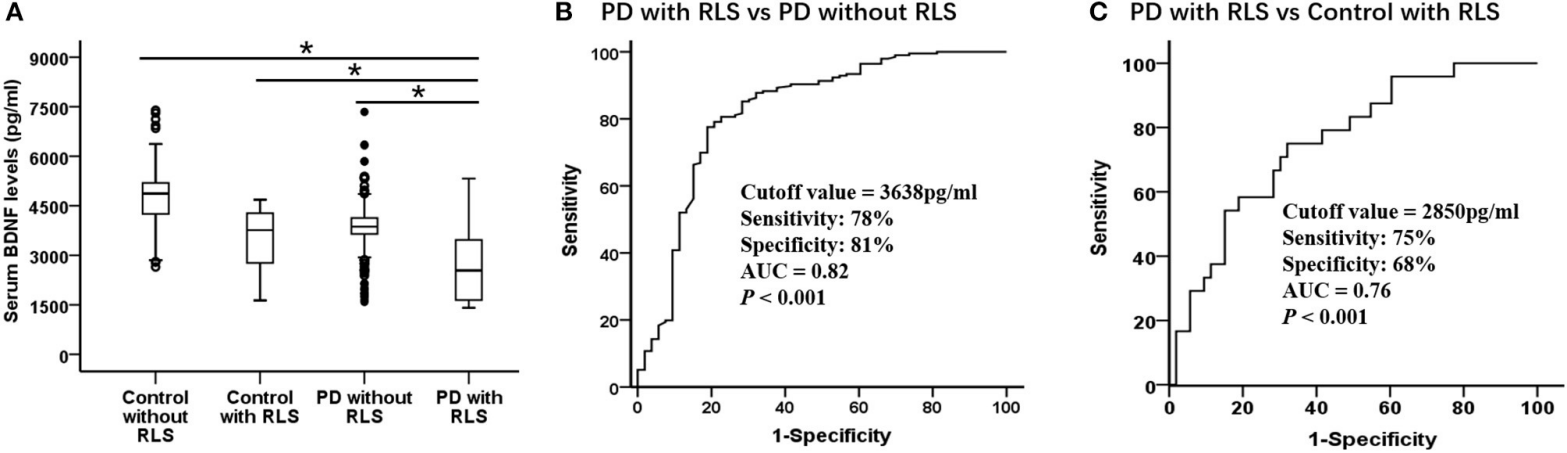
Serum BDNF highly discriminates PD with RLS from without RLS and controls. Serum BDNF concentration in control with RLS and without RLS, PD with RLS and without RLS and diagnostic accuracy. **(A)** Mean serum BDNF level was significantly decreased in PD with RLS compared with without RLS and control with RLS and without RLS. Mean levels were shown with SD; ^*^p < 0.001. **(B,C)** Receiver operating characteristic curve analyses for differentiating between **(B)** PD patients with RLS and without RLS and **(C)** PD patients with RLS and control with RLS. AUC, area under the curve; BDNF, brain-derived neurotrophic factor; PD, Parkinson's disease; RLS, restless legs syndrome.

As shown in Table 1 and Figure 1A, the serum BDNF levels in PD patients with RLS was significantly lower than that in PD patients without RLS, controls without RLS, and controls with RLS (PD with RLS, 2604.40 ± 1011.22 pg/ml; PD without RLS, 3814.57 ± 835.38 pg/ml; controls without RLS, 4779.18 ± 771.59 pg/ml; controls with RLS, 3502.63 ± 904.08; *p* < 0.001; effect sizes = 1.30, 2.42, and 0.94, respectively). After controlling for sex, age, education, BMI, alcohol, and smoking status, this significant difference still existed (*p* < 0.001). Furthermore, BDNF levels in both PD patients without RLS and controls with RLS were lower than in controls without RLS (both *p* < 0.001; effect sizes = 1.20 and 1.52, respectively). These two differences remained significant after controlling for sex, age, education, BMI, alcohol, and smoking (both *p* < 0.001). Furthermore, the ROC analysis showed that a serum BDNF level cutoff value of 2,850 pg/ml had a sensitivity of 75% and a specificity of 68% for discrimination of PD with RLS from control with RLS, with an area under the curve of 0.76 (Figure 1C). Because serum BDNF was lower in PD patients with RLS than without RLS (*p* < 0.001, Figure 1A), the serum BDNF cutoff value of 3,638 pg/ml had a 78% sensitivity and an 81% specificity for distinguishing between PD patients with RLS and without RLS, with an area under the curve of 0.82 (Figure 1B).

In addition, correlation analysis showed no significant correlation between BDNF and the disease duration, age of onset, H-Y stage, UPDRS score, and LED in either of PD patients with the RLS group, or in the PD patients without the RLS group (both *p* > 0.05). As shown in Table 2, PD patients were classified as having the TD and PIGD motor subtypes. There were no significant differences in demographic and BDNF level variables in PD with and without RLS (all *p* > 0.05). In our study, it is worth noting that the average H-Y stage (1.60 ± 0.82) and UPDRS III score (38.29 ± 14.43) of the whole PD patients in Table 1 were quite low, and the duration of the disease (4.3 ± 2.4 years) was relatively short.

**Table 2:** Comparison between TD-PD and PIGD-PD motor subtypes on demographic details and BDNF variables.

	**PD without RLS**			**PD with RLS**		
	**TD** **(***n*** = 139)**	**PIGD** **(***n*** = 57)**	***P*** **-value**	**TD** **(***n*** = 38)**	**PIGD** **(***n*** = 15)**	***P*** **-value**
Sex (M/F)	63/76	31/26	0.249	17/21	6/9	0.754
Age (years)	62.24 ± 9.51	60.46 ± 8.21	0.216	62.84 ± 9.87	62.73 ± 10.45	0.972
Education (years)	9.26 ± 1.95	8.93 ± 1.83	0.277	9.13 ± 1.93	9.80 ± 2.37	0.293
BDNF (pg/ml)	3792.73 ± 674.57	3867.82 ± 1142.18	0.644	2617.95 ± 993.86	2570.07 ± 1088.95	0.878

*Data are presented as the mean ± standard deviation (mean ± SD)*.

*BDNF, brain-derived neurotrophic factor; PD, Parkinson's disease; PIGD, postural instability/gait disturbances dominant; RLS, restless legs syndrome; TD, tremor dominant*.

*Parameters were analyzed with analysis of variance using Bonferroni as post-hoc test in the case of normal distribution of data. For variables that did not display a normal distribution, data were compared between the TD-PD and PIGD-PD groups by Mann–Whitney U-test. Sex was analyzed using χ^2^-test*.

### Correlation Between Serum Brain-Derived Neurotrophic Factor Level and Restless Legs Syndrome Severity

**Figure 2:**
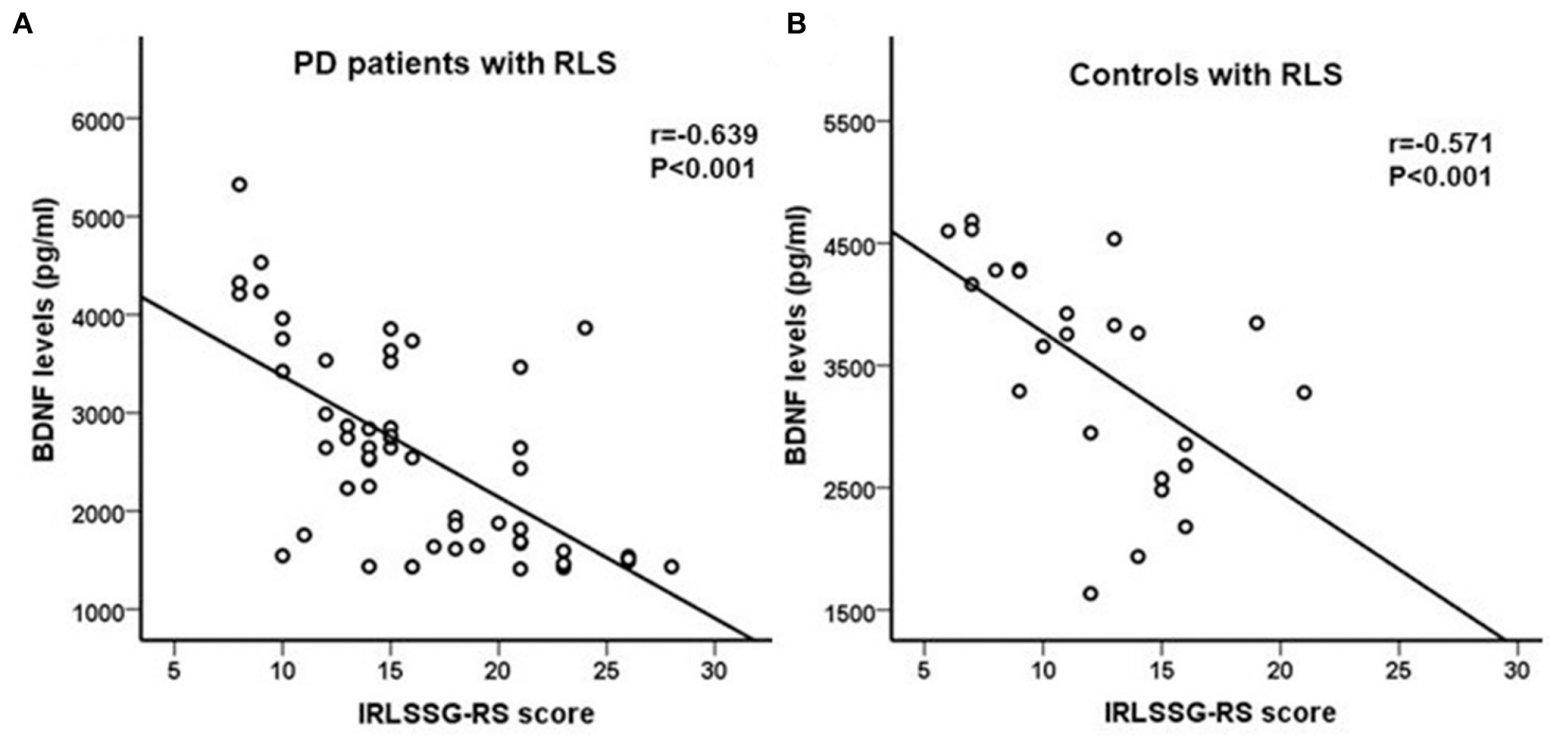
**(A,B)** Correlation analysis showed a significant negatively association between serum BDNF levels and IRLSSG-RS score in PD patients with RLS and in controls with RLS (both *p* < 0.001). BDNF, brain-derived neurotrophic factor; IRLSSG-RS, International Restless Legs Syndrome Study Group Rating Scale; PD, Parkinson's disease; RLS, restless legs syndrome.

In the PD patients with the RLS group, correlation analysis showed that BDNF levels were significantly negatively correlated with IRLSSG-RS score (*r* = −0.639, df = 51, *p* < 0.001; Bonferroni corrected *p* < 0.01; Figure 2). In the controls with the RLS group, correlation analysis also showed a significantly negative association between BDNF and the IRLSSG-RS score (*r* = −0.571, *df* = 22, *p* < 0.001; Bonferroni corrected *p* < 0.01; Figure 2). Further multivariate regression analysis showed that the correlation between serum BDNF levels and IRLSSG-RS scores remained significant in either PD with RLS or controls with RLS group (*t* = −5.764, *p* < 0.001; Table 3; *t* = −2.968, *p* = 0.008; Table 4), while adjusting for potentially confounding demographics, including sex, age, education, and BMI. These findings suggested that lower serum BDNF levels were correlated with higher RLS severity scores in PD with RLS and controls with RLS.

**Table 3:** Multivariate regression models for RLS severity assessed by IRLSSG-RS scores in PD patients with RLS.

**Independent variables**	**Coefficient**	**Standard error**	***r*** **value**	***t*** **value**	***p-*** **value**
Constant	25.115				
Sex	0.490	1.259	0.047	0.389	0.699
Age	0.053	0.060	0.100	0.880	0.383
Education	0.168	0.292	0.066	0.574	0.569
BMI	−0.240	0.284	−0.095	−0.844	0.403
BDNF level	−0.003	0.001	−0.657	−5.764	<0.001^a^

*BMI, body mass index; BDNF, brain-derived neurotrophic factor; IRLSSG-RS, International Restless Legs Syndrome Study Group Rating Scale; PD, Parkinson's disease; RLS, restless legs syndrome*.

*In this model, the dependent variable was the IRLSSG-RS score, and the independent variables were sex, age, education, BMI, and serum BDNF level. Baseline serum BDNF levels were included as continuous predictor variables. r is the correlation coefficient of the multiple regression; t is the coefficient of each parameter in the model*.

a *p < 0.05*.

**Table 4:** Multivariate regression models for RLS severity assessed by IRLSSG-RS scores in controls with RLS.

**Independent variables**	**Coefficient**	**Standard error**	***r*** **value**	***t*** **value**	***p-*** **value**
Constant	24.567				
Sex	−1.258	1.813	−0.161	−0.694	0.497
Age	0.015	0.061	0.052	0.248	0.807
Education	−0.307	0.506	−0.143	−0.607	0.552
BMI	0.001	0.356	0.001	0.003	0.998
BDNF level	−0.003	0.001	−0.569	−2.968	0.008^a^

*BMI, body mass index; BDNF, brain-derived neurotrophic factor; IRLSSG-RS, International Restless Legs Syndrome Study Group Rating Scale; PD, Parkinson's disease; RLS, restless legs syndrome*.

*In this model, the dependent variable was the IRLSSG-RS score, and the independent variables were sex, age, education, BMI, and serum BDNF level. Baseline serum BDNF levels were included as continuous predictor variables. r is the correlation coefficient of the multiple linear regression; t is the coefficient of each parameter in the model*.

a *p < 0.05*.

## Discussion

Previous studies have shown that dopamine system deficit plays a vital role in the pathophysiology of PD and RLS (14, 15) because of the frequent co-occurrence of PD with RLS, and the two disorders may both respond well to dopaminergic drugs (12). Different studies have demonstrated that RLS is more common in PD patients (10–50%) than in the general population (2.5–10%) (3, 7–11). Consistent with previous data, our findings revealed that the prevalence rates of RLS was higher in PD patients (21.3%) than in controls (7.5%). This result may corroborate for the hypotheses of similar pathophysiology between RLS and PD (14, 15). Furthermore, the IRLSSG-RS score in PD patients with RLS (16.25 ± 5.24) was significantly increased than that in controls with RLS (12.08 ± 3.99), indicating that patients with PD had more severe RLS.

It is known that PD leads to an almost 80% reduction in the DAergic neurons of substantia nigra pars compacta (33). Many studies have shown that BDNF and the midbrain DA systems interact with each other, and BDNF promotes the survival of DAergic neurons in the substantia nigra (22). Also, BDNF affects the release of DA in the mesolimbic DA system and the induction of DA-related behaviors (23). These studies suggest a close relationship between the BDNF and DA systems. Several lines of evidence reveal that the lower BDNF expression in the substantia nigra is associated with DA neuronal loss in PD animal models and patients compared with that in controls (20, 21). Recent studies have shown that rotigotine, as a non-ergot dopamine receptor agonist, is clinically used in the treatment of restless legs syndrome, which may play its role by improving the function of BDNF in the brain (34, 35). These findings suggest a close relationship between BDNF and RLS in PD patient. To clarify what insights can be drawn from the available evidence, we conducted the first study on the relationship between decreased BDNF levels and risk of RLS in PD.

In the present study, serum BDNF levels were significantly decreased in PD patients with RLS than without RLS, controls with RLS and without RLS. Consistent with our data, the decreased levels of serum BDNF in PD patients have been observed in previous studies (21). The ROC analysis showed that the serum BDNF had a 78% sensitivity and an 81% specificity (AUC 0.82) in distinguishing between PD patients with RLS and without RLS, and had a 75% sensitivity and a 68% specificity (AUC 0.76) in distinguishing between PD with RLS and control with RLS. Taken together, these findings suggest that reduced BDNF may play an important role in the pathogenesis of PD patients with RLS, and it may be a useful clinical biomarker in differentiating PD patients with RLS from without RLS and controls.

In view of the decreased BDNF levels, which may be associated with pathological alterations of the DAergic neurons in PD, and the important role of decreased DAergic neurons in the development of RLS, it would be of interest to explore the association between RLS and BDNF in PD. To the best of our knowledge, none of the previous studies has assessed the correlation in PD. Our results showed that BDNF levels were negatively correlated with IRLSSG-RS in both the PD patients with RLS and the controls with RLS. According to previous studies, we speculated that the decrease in BDNF in the RLS control group is related to the reduction of DAergic neurons (15, 34, 35). Further multiple regression analysis confirmed that in either PD with RLS or controls with the RLS group, BDNF was an independent contributor to IRLSSG-RS. Our results suggest that decreased serum BDNF levels may serve as a potential biomarker of diagnostic value for RLS in PD patients.

This study suffers from several limitations. First, the study design was cross-sectional, which limits causal inference between clinical features of PD and BDNF. Second, we did not collect some more important clinical data, such as family history, coffee intake, hypertension, coronary heart disease, and other information, which may also affect the RLS in PD patients. Third, our RLS prevalence estimates are not generalizable for the PD population due to the stratification procedure in our study, mostly patients with mild-to-moderate PD are referred to. Fourth, all patients were under levodopa therapy, which could affect both prevalence and severity of RLS. Fifth, the exclusion of PD patients beyond levodopa monotherapy may not represent the whole PD population. Sixth, our clinical trial population was entirely Han Chinese and lacked ethnic diversity. Other limitations in the clinical trial include the serum BDNF level at single time point, rather than at multiple time points.

In conclusion, serum BDNF levels were lower in PD patients with RLS than in PD patients without RLS, in controls without RLS and in controls with RLS. Correlation analysis showed that BDNF levels were negatively correlated with IRLSSG-RS scores in PD patients with RLS and controls with RLS. Interestingly, further multiple regression analysis confirmed that in either PD with RLS or controls with RLS group, BDNF was an independent contributor to IRLSSG-RS. Collectively, our results suggested that decreased serum BDNF levels may be involved in the pathophysiology of RLS in PD patients, and it may serve as a potential blood biomarker of diagnostic value for RLS in PD patients.

## Data Availability Statement

The original contributions presented in the study are included in the article/supplementary material, further inquiries can be directed to the corresponding author/s.

## Ethics Statement

The studies involving human participants were reviewed and approved by the Second Affiliated Hospital of Soochow University. The patients/participants provided their written informed consent to participate in this study. Written informed consent was obtained from the individual(s) for the publication of any potentially identifiable images or data included in this article.

## Author Contributions

Y-xH, Q-lZ, and C-lH designed the research. Y-xH, Q-lZ, J-wS, and W-qW performed the research. Y-xH, Q-lZ, and C-lH analyzed the data. All authors have read and approved the final manuscript.

## Funding

This work was supported by the National Natural Science Foundation of China (Grant No. 81200970), the Suzhou Sci and Tech Program (Grant No. SYSD2018098), and the Changzhou High-Level Medical Talents Training Project (Grant No. 2016CZBJ023).

## Conflict of Interest

The authors declare that the research was conducted in the absence of any commercial or financial relationships that could be construed as a potential conflict of interest.

## Publisher's Note

All claims expressed in this article are solely those of the authors and do not necessarily represent those of their affiliated organizations, or those of the publisher, the editors and the reviewers. Any product that may be evaluated in this article, or claim that may be made by its manufacturer, is not guaranteed or endorsed by the publisher.
